# Janus-Faced Myeloid-Derived Suppressor Cell Exosomes for the Good and the Bad in Cancer and Autoimmune Disease

**DOI:** 10.3389/fimmu.2018.00137

**Published:** 2018-02-02

**Authors:** Margot Zöller

**Affiliations:** ^1^Tumor Cell Biology, University Hospital of Surgery, University of Heidelberg, Heidelberg, Germany

**Keywords:** myeloid-derived suppressor cells, exosomes, cancer, autoimmune disease, therapy

## Abstract

Myeloid-derived suppressor cells (MDSCs) are a heterogeneous population of immature myeloid cells originally described to hamper immune responses in chronic infections. Meanwhile, they are known to be a major obstacle in cancer immunotherapy. On the other hand, MDSC can interfere with allogeneic transplant rejection and may dampen autoreactive T cell activity. Whether MDSC-Exosomes (Exo) can cope with the dangerous and potentially therapeutic activities of MDSC is not yet fully explored. After introducing MDSC and Exo, it will be discussed, whether a blockade of MDSC-Exo could foster the efficacy of immunotherapy in cancer and mitigate tumor progression supporting activities of MDSC. It also will be outlined, whether application of native or tailored MDSC-Exo might prohibit autoimmune disease progression. These considerations are based on the steadily increasing knowledge on Exo composition, their capacity to distribute throughout the organism combined with selectivity of targeting, and the ease to tailor Exo and includes open questions that answers will facilitate optimizing protocols for a MDSC-Exo blockade in cancer as well as for strengthening their therapeutic efficacy in autoimmune disease.

## Introduction

### Myeloid-Derived Suppressor Cells (MDSCs) and Cancer

Cancer is one of the most frequent causes of death (1), which in part is due to the resistance of tumor cells to chemo-, radio-, and immunotherapy (2–4). This implies that after tumor spread, which might prohibit surgical excision, the likeliness of curative therapy steeply declines. Disappointing efficacy of adjuvant cancer therapies accounts particularly for immunotherapy, where frequently no or only weak responses are noted despite the presence of immunogenic tumor-associated antigens (5). In several tumor entities, MDSCs were found to account for resistance toward cancer immunotherapy (6) and additionally for poor responses to chemotherapy. Therefore, drugs were designed that, besides directly attacking the tumor cells, should hamper MDSC development or activation or drive MDSC into apoptosis (7). So far therapeutic trials with a focus on MDSC elimination to improve chemotherapy or immunotherapy are rare, which also accounts for combinations of adjuvant therapeutics (8, 9). The current options and possible modes of improvement attacking MDSC/MDSC-exosomes (MDSC-Exo) to support cancer chemo- and/or immunotherapy will be discussed.

### MDSC in Autoimmune Disease and Allograft Transplantation

Autoimmune disease incidence is steadily increasing (10). Autoimmune diseases frequently exacerbate in young adults and progress in waves, which get more severe during time and become life threatening (11). Corticosteroid therapy, frequently used in progressed disease stages (12), is burdened by severe side effects, including dampening immune responses against bacteria and viruses (13). The option to booster autoimmune disease therapy with MDSC (14) gained in weight, when it was realized that MDSC are a strong stimulus for regulatory T cell (Treg) activation, a deficit in Treg contributing to autoreactive T cell expansion (15). There are several trials to integrate MDSC in autoimmune disease therapy, such as myasthenia gravis, arthritis, inflammatory bowel disease, and others, where good response rates were reported (14, 16–21).

Myeloid-derived suppressor cell-promoted downregulation of immune reactivity also is advantageous in allograft transplantation. This accounts for organ as well as hematopoietic stem cell (HSC) transplantation (22–26). Accordingly, drugs promoting MDSC expansion and/or activation and the transfer of MDSC were reported to support long-term allograft survival (27–30).

Having introduced the two faces of MDSC, this review will focus on MDSC and MDSC-Exo in cancer and autoimmune disease. After introducing MDSC and Exo, their mode of action in disease will be outlined. Knowledge on the crosstalk between MDSC/MDSC-Exo and their targets provides the fundament for established and forthcoming therapeutic interference.

## MDSC: Phenotypic and Functional Characterization

Myeloid-derived suppressor cells are a heterogeneous group of cells, characterized by myeloid origin, immature state, and mostly functional activity. In humans, MDSC are still difficult to isolate due to an inconclusive surface marker expression profile. However, there is consent on the differentiation between two subgroups defined as monocytic (M) and granulocytic MDSC (G-MDSC), which are differentiated on the basis of Ly6C high [monocytic MDSC (M-MDSC)] or Ly6G high (G-MDSC), M-MDSC exerting stronger suppressive activity (31–33). MDSC account for T cell exhaustion in chronic infections (34, 35), play a crucial role in cancer progression (31, 36), and are a major hindrance in cancer immunotherapy, hampering T cell recruitment and activation, while promoting M1 and Treg expansion (14, 37). On the other hand, MDSC are beneficial in overshooting immune reactions such as autoimmune diseases (24, 33) and allogeneic transplantation (18, 33, 38). Finally, though the activity of MDSC may vary with the pathophysiological conditions promoting their expansion, there is consent that T cells are major targets and that the response of the adaptive immune system is most severely affected (39).

Myeloid-derived suppressor cell expansion is promoted by lipopolysaccharide, macrophage colony stimulating factor, GM-CSF, SCF, IL6, interferon (IFN)γ, IL1β, vascular endothelial growth factor (VEGF), heat shock protein (HSP)72, IL13, C5a, PGE2, and S100A8/A9 (32, 40). Downstream activation of the JAK– signal transducer and activator of transcription (STAT)3/STAT5 pathway with stimulation of cyclinD1, Bcl2-like (BclXl), survivin, c-myc, and S100A8/A9 contribute to inhibition of differentiation into mature myeloid cells. MDSC recruitment is supported by provision of CCL2, C-X-C motif chemokine ligand (CXCL)12, and CXCL15, the corresponding ligands being expressed by MDSC. Intracellular molecules involved in MDSC function are STAT3, COX2, HIF1α, C/EBPβ, inducible nitric oxide synthase (iNOS), arginase (Arg), HO-1, and indoleamine 2,3-dioxygenase (IDO) (32, 40, 41). Downstream effector molecules are Arg-1 and iNOS. Arg-1 and iNOS account for depletion of l-arginine in T cells, which contributes to ζ-chain downregulation. iNOS induces NO, NO and ROS inhibiting T cell proliferation and inducing apoptosis. HO-1 inhibits T cell proliferation *via* CO production. Membrane-bound TGFβ1 promotes natural killer cell (NK cell) anergy. IL10 and TGFβ foster Treg expansion (40), which become recruited by CXCL10. TGFβ, and IL10 also account for downregulation of IFNγ (40). IL10 promotes TH2 deviation and type 2 macrophage (Mϕ) polarization. Finally, ADAM17 leads to CD62L cleavage, which prohibits T cell homing (42, 43).

In cancer, drugs have been developed and are further improved to selectively attack MDSC maturation and/or activation. These include, besides others, all-transretinoic acid (ATRA) driving progenitors into differentiation, the tyrosine kinase inhibitor sunitinib, gemcitabine, COX-2 inhibitors, and the phosphodiesterase-5 inhibitor sildanefil (44–48). In autoimmune diseases and allogeneic bone marrow (BM) transplantation, the transfer of MDCS was demonstrated as a therapeutic option (18, 33, 38, 42, 49).

Taken together, MDSC are immature myeloid cells that hamper mostly T cell, but also B cell and NK activity, at least in part by supporting Treg expansion and activation. They are a severe hindrance in cancer immunotherapy and in chronic infections. Mostly in cancer immunotherapy drugs and drug combinations to prevent MDSC induction, activation and targeting as well as drugs to drive MDSC into apoptosis are experimentally and clinically explored to improve the efficacy of immunotherapy. Based on the same principle MDSC activity is suited to control undesired immunoreactivity in transplantation and autoimmune disease, the transfer of MDSC being a therapeutic option.

## Exosomes (Exo)

Exosomes are small 40–100 nm vesicles delivered by most cells of an organism (50). They distribute throughout the body and are recovered in all body fluids (51). Exo express donor cell-derived components. This finding stimulated Exo research as a non-invasive/minimally invasive tool for diagnosis, prognosis and therapy control (51, 52). Of particular importance was the notion that Exo components are function competent and deliver their messages into target cells (53, 54) such that Exo binding and uptake can severely modulate target structures and suffices for reprogramming target cells (54–57). Furthermore, Exo easily can be modulated *in vitro* (58). Thus, Exo are a most powerful intercellular communication system and are supposed to become a highly effective therapeutic tool in the near future (59, 60).

### Exo Biogenesis

Exosome biogenesis starts with the formation of early endosomes (EE), which can derive from the trans-Golgi network or from different internalized membrane microdomains, such as clathrin-coated pits, tetraspanin and glycolipid-enriched membrane domains (GEM), or proteolipids in cholesterol- and ceramide-rich compartments (61). EE move toward multivesicular bodies (MVB), the transport machinery varying for the different types of EE (62). During inward budding of EE into MVB, called intraluminal vesicles (ILV), vesicles receive their cargo. Loading of the small plasma that could contain ~100 proteins and 10,000 nucleotides (63) with proteins, coding and non-coding RNA and DNA are non-random processes (61). Sorting of proteins is facilitated by mono-ubiquitination, acylation or myristoylation (64, 65). For GEM-derived Exo, higher order oligomerization is important (66), where protein complexes and attached cytoplasmic components are retained (67). In raft-derived ILV, sphingolipids forming ceramide also contribute to vesicle loading (68). miRNA recruitment is guided by a zip code in the 3′-UTR and by coupling of RNA-induced silencing complex to components of the sorting complex. A specific EXOmotif (GGAG) controls miRNAs loading by binding to the heterogeneous ribonucleoprotein A2B1 (hnRNPA2B1), which binds to an RNA transport signal (A2RE) (69). Annexin-II plays a role in RNA sorting into ILV by binding specific RNAs (70). lncRNA also are selectively recruited by so far unknown mechanisms (71). Ras-related proteins regulate MVB movement toward the cell membrane (72). MVB fuse with the plasma membrane, ILV are released and are then called Exo (61).

Though there remain open questions on the precise biogenesis pathways, it is important to remember that due to differences in biogenesis, single cells can deliver different Exo (73, 74). For judging on potential diagnostic and therapeutic validity, information on the Exo composition is a prerequisite.

### Exo Composition

Exosomes are composed of a lipid bilayer, which contains transmembrane proteins. The intravesicular content is composed of proteins, coding and non-coding RNA and DNA.

The lipid envelop of Exo contains phosphatidylcholine, phosphatidylethanolamine, phosphatidylinositol, prostaglandins, and lysobisphosphatidic acid and is enriched in sphingomyelin, cholesterol, GM3, and phosphatidylserine (75). The high phosphatidylserine content allows differentiating Exo from microvesicles (76) and tumor-derived Exo (TEX) lipid composition may be suited for diagnosis (77, 78). Progress in lipidomics will provide further informations.

Improvement in mass spectrometry (79) has greatly facilitated the characterization of Exo proteins, where >7,000 were identified so far (80). Constitutive Exo proteins are structural vesicle component or are involved in vesicle biogenesis and vesicle trafficking. Most abundant are tetraspanins (81), enriched 7- to 124-fold in Exo compared to the parental cells (82). Adhesion molecules, proteases, MHC molecules, HSPs, TSG101, Alix, annexins, cytoskeleton proteins, metabolic enzymes, cytosolic signal transduction molecules, and ribosomal proteins, some of which are recruited *via* their association with proteins engaged in biogenesis, are also abundantly recovered (83, 84). Cell type-specific Exo proteins are so far most comprehensively explored for cancer and cancer stem cells (CSC), such as MART1, EGFRVIII, multidrug resistance gene 1, EpCAM, MET, mutant KRAS, and tissue factor (73, 85–88). Notably, due to their location in internalization prone microdomains, all known CSC markers are recovered in TEX (89, 90), which implies recovery of CSC marker expressing Exo in body fluids as most reliable for diagnosis.

Next generation sequencing allowed for rapid progress in Exo DNA, coding and non-coding RNA identification (91). Previous studies were mostly concerned about miRNA, which constitutes only 1–3% of the human genome, but due to multiple targets, controls about 30% of the coding genes. miRNA cleaves mRNA *via* argonaute (AGO) (perfect base pairing) or represses translation (imperfect binding) (92). Knowledge on miRNA greatly fostered progress in oncology, where miRNA could be linked to prognosis, disease progression, local recurrence, and metastasis (93), particularly the miR-200 family playing an important role in epithelial–mesenchymal transition (EMT) (94). miRNA also accounts for CSC maintenance (95), angiogenesis (96), and chemoresistance (97). Other miRNA, such as miR-34, -34a, -340, act as tumor and metastasis suppressors (98, 99).

miRNA also regulates tolerance induction and inflammation (100–102). A knockout of Dicer and Ago2 in HSC results in increased apoptosis and loss of hematopoietic cell reconstitution; Ago2 deletion is accompanied by deficient B and erythroid cell differentiation (103). A knockdown of Dicer results in T cell reduction (104, 105) and a shortened survival rate and reduced antibody repertoire in B cells (106). Dicer and Drosha also are required for Treg regulation (107, 108), a ko promoting a lethal inflammatory disease. MiR-155 regulates NK maturation and activation by suppressing suppressor of cytokine signaling 1 and phorbol-12-myristate-13-acetate-induced protein 1 (109). In MDSC, upregulated miR-494 and -21 target phosphatase and tensin homolog (Pten), miR-155 targets Ship-1 and miR-210 Arg1, whereas downregulated miR-17-5p and -20a target STAT3 (110). In asthma, miR-20b promotes G-MDSC accumulation associated with a decrease in IL-3 and IL-13 (111) and miR-223 suppresses Arg1 and STAT3 in multiple sclerosis and autoimmune encephalitis (112). Thus, miRNA, besides being important in oncogenesis and tumor progression, regulates T cells, B cells, and components of the innate immune system including MDSC, which has severe bearing on inflammation and autoimmune diseases.

Long non-coding (lnc)RNA makes up ~3% of total exosomal RNA and is transferred into host cells (113). Several exosomal lncRNAs, such as MALAT-1, linc-POU3F3, ZFAS1, promote tumor growth and migration, prevent tumor cell apoptosis, or induce angiogenesis (114–117). A most comprehensive study on colorectal cancer revealed 1,028 lncRNAs selectively enriched in Exo, where the co-existence of U1 and U2 rib nucleoproteins and their cognate shrines in the Exo suggests a possible link to recipient cell splicing events (118). Exosomal lncRNA (GAS5) also regulates apoptosis of Mϕ (119), hematopoietic, innate, and adaptive immune responses (120, 121). The recovery of exosomal lncRNA and novel splicing/fusion genes will be important in developing Exo-based therapeutics.

Taken together the ongoing analysis of Exo composition provided a plethora of informations, which strongly sustain the initial hypothesis of Exo as important intercellular communicators allowing sessile cells a systemic communication, which is equally important in physiology and pathology. For optimal therapeutic translation, further analyses with a focus on donor-dependent differences in Exo profiles are desirable.

### Exo Targeting and Uptake

Answering the questions how Exo find their targets and are there options to guide targeting is urgent for therapeutic considerations (122). Exo can bind to the extracellular matrix (ECM) or cells *via* specific receptor–ligand pairs, where binding accounts for matrix and cell modulation (123, 124). Exo uptake also depending on target cell ligands, may require different target structures then binding and can have distinct consequences for the target cell (125, 126).

Exosomes bind to and are taken up by selected target cells. Exo binding frequently involves (tetraspanin-associated) integrants (124, 127), where ICAM1 will be one potential partner (128, 129). Notably, different integrants bind distinct target cells. Thus, the α6β4 integrant binds cells in the premetastatic niche of the lung, whereas integrin αvβ5 binds cells in the premetastatic niche of the liver (127). A Tspan8–α4β1 complex binds to endothelial cells (EC) and EC progenitors, but a Tspan8–α6β4 complex hampers Exo uptake by EC (130). Other known binding partners are proteoglycans prevalently binding to galectins, selectins, and sialic acid binding lectins (131–134). According to our experience, Exo binding is greatly facilitated by clusters of adhesion molecules in both Exo and target cells (84).

Exosome binding mostly is followed by uptake. There are two modalities for Exo uptake, fusion with the cell membrane (135, 136) and, dominating, endocytosis, an active process that requires modulation of the actin cytoskeleton (135, 137–139). There are at least four modes of uptake, phagocytosis, macropinocytosis, clathrin-dependent endocytosis, and uptake by lipid rafts and caveolae. Phagocytosis proceeds *via* the formation of cup-like extensions, where the tips fuse and become internalized. Phagocytic markers like lysosomal-associated membrane protein 1 on Exo (140) and T-cell immunoglobulin and mucin domain containing (TIM)4 that recognizes phosphatidylserine on Exo facilitate the process (136, 137, 141). Exo uptake by macropinocytosis occurs, when lamellipodia fold back and fuse with the plasma membrane (142, 143). Most frequently, Exo endocytosis proceeds *via* clathrin-coated pits, where dynamin contributes to the scission of clathrin-coated endocytosed pits (84, 139, 140). Finally, Exo can be internalized by rafts, cholesterol-, and glycolipid-enriched membrane microdomains, such as tetraspanin webs (84, 139, 144) or caveolae (145).

In brief, Exo uptake is an active process with a contribution of the cytoskeleton as well as fission and scission machineries to detach from the plasma membrane. Intracellular processing of the uptaken Exo varies between cells and requires further exploration (146). Though Exo may itinerate (147), they mostly are digested, their content modulating the target cell both directly or by stimulating signaling cascades, transcription, and silencing processes *via* the target cell’s equipment (148–151).

### Exo and Target Cell Reprogramming

Whether Exo-induced changes in target cells are due to the transferred content of Exo or to transfer-induced target cell reactions is still disputed. When Exo bind to the ECM, the Exo membrane-coat accounts for changes observed in matrix proteins and matrix structure. Instead, when Exo bind to, but are not taken up by the target cell, target cell modulation is promoted by Exo-initiated signal transduction and/or cleavage of proteins on the target cell membrane. When Exo are taken up by the target cell, an unequivocal answer is more critical. There are examples, demonstrating that changes in the target cell are directly due to the transferred Exo content. Thus, in prostate cancer cells, αvβ6 is transferred *via Exo* into an αvβ6-negative recipient cell and localizes to the cell surface, *de novo* αvβ6 expression by the recipient cell being excluded (152). Also, after dendritic cell (DC) loading with TEX, tumor antigens are processed and loaded into newly synthesized MHC molecules (137, 153). The same principle will be valid for therapeutically tailored Exo loaded with large amounts of therapeutics drugs or miRNA or signaling checkpoint inhibitors (154–156). However, whether the naturally available amount of one type of Exo contains sufficient load to directly modulate targets is questionable. First, the small Exo plasma homes a limited amount of proteins and nucleotides; second, a TEX preparation from a cloned tumor line distinctly affects tumor cells, fibroblasts, EC, and hematopoietic cells. Thus, an impact on signal transduction and/or transcription/translation likely represents the dominating mode of uptaken Exo activity. The strong impact of DC-Exo uptake by the immune synapse also supports an initiator role of the transferred Exo content (157, 158). The hypothesis is backed by activation or inhibition of B cells, NK, and neutrophils initiated by DC-, Mϕ-, stem cell (SC)-, or tumor cell-derived Exo (159–163). The important role of Exo for anterograde and retrograde information transfer *via* neurological synapses also argues for Exo-initiated activation of signal transduction, where Exo-promoted activation of signaling cascades was described to maintain plasticity under physiological conditions as well as to account for the spread of pathological proteins (164–167).

Thus, without excluding target modulation by uptaken Exo content, in most instances an incentive push by Exo superiorly covers the wide range of Exo activities.

### Exo Transfer and the Life Span of Exo

Information on the natural life span and that of transferred Exo is an additional prerequisite for therapeutic trials.

“Therapeutic” Exo recovery in serum after intravenous application declines toward ~50% within 2 min–1 h (168, 169). Instead, uptaken Exo are recovered for several days (170–172). Thus, gold-labeled Exo could be tracked for over 24 h after intranasal or i.v. application (173) and were recovered in liver, lung, BM, peripheral blood leukocytes, and spleen cells for up to 48 h, with particularly high recovery in monocytic cells, including MDSC (168, 169).

Where required, therapeutic rescuing can be further improved by tailoring with docking molecules (174, 175). Tetraspanins and RGD peptides were described to target tumor cells or EC (176, 177). Targeting oncogenic receptors or SC receptors offers an alternative strategy (178). Bacterial-derived extracellular mimetics additionally could facilitate generating large quantities of homogeneous Exo for vaccination and drug delivery (179).

Taken together, available data strongly support the feasibility of therapeutic Exo application to interfere with cancer progression, to balance angiogenesis, blood coagulation, and to regulate native or adaptive immune system responses. MDSC-Exo are engaged in all these processes.

### MDSC-Exo Characterization

Myeloid-derived suppressor cells are well characterized and there is a wealth of information on the impact of TEX on MDSC. Information on MDSC-Exo is limited and was mostly collected using MDSC-Exo derived from tumor-induced, immunosuppressive MDSC, which resemble the inflammatory MDSC in chronic infections. Thus, these data are valid for the differentiation between resting versus inflammatory MDSC-Exo in general.

Myeloid-derived suppressor cell-exosomes contain common Exo components such as annexins, tetraspanins, glycosylphosphatidylinositol-anchored CD177, cytoskeletal proteins, proteins engaged in vesicle biogenesis, and HSP. There is an abundance of proteasome subunits, histone variants and elongation factors, and metabolic enzymes that recovery in MDSC-Exo mostly corresponds to the recovery in MDSC. Comparing inflammatory with conventional MDSC-Exo showed a decrease of 33 proteins, some of which being involved in innate immune responses, such as complement components and chemotactic proteins. In addition, some cytoskeletal proteins like spectrin, ankyrin, and tubulin were reduced in inflammatory MDSC-Exo. Thirty proteins increased in inflammatory MDSC-Exo included GTP and ATP-binding proteins and proteins engaged in Exo-biogenesis facilitating budding or sorting (180).

The same group also reported on the abundance of ubiquitinated proteins in MDSC-Exo, a posttranslational modification that contributes to internalization of membrane proteins and the sorting of endosomal proteins (181), which also accounts for five newly recovered ubiquitinated proteins [sortin nexin 13, two keratins (krts), leucine zipper, and EF-hand containing transmembrane protein 1 < LETM1 > and endoplasmin] (182). Furthermore, MDSC-Exo abundantly carry ubiquitinated histones, the non-histone nuclear protein high mobility group box (HMBG)1 as well as all the enzymes required to catalyze ubiquitination (183). A proteome analysis of low mass proteins in MDSC-Exo confirmed, besides the abundance of proinflammatory S100 proteins the abundance of histones, which made up 56% of the MDSC-Exo protein cargo (184). Of special interest also is the analysis of MDSC-Exo surface glycoproteins, which include fibronectin (FN), olfactomedin4, galectin-3-binding protein, myeloperoxidase, thrombospondin1 (Tsp1), a cytoskeletal krt (Krt77), fibrinogen (Fbg), mast cell expressed protein 1, transmembrane 9 superfamily member 3, endothelial lipase, and the CD molecules CD44, CD157, CD11b, CD97, CD39, CD18, CD321, CD41 (185). Searching for potential ligands revealed several shared ligand receptor pairs, like CD41: Tsp, FN, Fbg; CD11b: haptoglobin (Hp), FN, Fbg; CD18: Hp, Fbg; CD47: Tsp, CD172a (SIRPa); CD44: Tsp, CD29, CD47, indicating that MDSC-Exo are well equipped for binding. However, there were no hints toward a pronounced selectivity of binding. Instead, several of these MDSC-Exo membrane proteins could be of functional interest. The do not-eat me CD47, whose dominating ligand is Tsp1, promotes MDSC migration (186). Furthermore, MDSC express the advanced glycosylation end-product specific receptor ligand S100A8/9, which could contribute to the activation of inflammatory/immunosuppressive genes (185–187).

Information on the RNA and DNA load of MDSC-Exo is largely missing, but is well explored in MDSC (188, 189). To give a few examples, TGFβ promotes G- and M-MDSC induction and expansion *via* upregulation of miR-155 and miR-21, which target inositol phosphate-5-phosphatase D and Pten, leading to activation of STAT3 (190). During sepsis, miR-21 and miR-18b become strikingly upregulated, which is accompanied by pronounced immunosuppressive activity of MDSC prohibiting bacterial clearance (191). Mir-9 overexpression enhances MDSC functional activity. This is due to miR-9 targeting Runx1, an essential transcription factor in promoting MDSC differentiation (192). Doxorubicin treatment promotes miR-126a induction in MDSC. miR-126a + MDSC-Exo induce IL13+Th2 cells and rescue MDSC death in a S100A8/A9-dependent manner (193).

Taken together, the inflammatory MDSC-Exo membrane protein profile provides hints toward receptor–ligand pairs. Unfortunately, so far no selective ligands, e.g., for binding Treg or activated T cells were recovered. The reduced recovery of some inflammatory proteins in inflammatory MDSC-Exo suggests a possible contribution to the inefficacy of immune-response induction in cancer and chronic infections. The abundance of proteasome subunits as well as of histones and HMBG1, which is inflammation independent, is of great interest and should be further elaborated, some functional consequences being already defined. The finding that Doxorubicin treatment affects the MDSC-Exo miRNA profile with severe functional consequences also should spur research on this topic.

## Coping with MDSC-Exo in Cancer

### MDSC-Exo Activities in Cancer

As Exo are supposed to be most important intercellular communicators, can be easily modulated *in vitro* and are simple to store for therapeutic application, a detailed knowledge on MDSC-Exo activities will open a wide range of new and promising therapeutic applications. However, gaining insight is a demanding task. This relates to the heterogeneity of MDSC, the delivery of distinct Exo subpopulations by individual cells, the differences in Exo delivered by MDSC during maturation in the BM versus “inflammatory” MDSC. In addition, Exo have more than one target, which becomes aggravated by the distribution of Exo throughout the body and the cooperativity of different cells/subpopulations particularly in the immune system. This implies that a whole range of potential targets needs to be analyzed for MDSC-Exo promoted alterations.

Though not directly approaching MDSC-Exo, there is compelling evidence that TEX induce and affect MDSC. TEX are taken up by myeloid cells in the BM and switch their differentiation toward MDSC. Also, tumor growth-promoting activity of MDSC depends on PGE2 and TEX-provided TGFβ that induce upregulation of Cox2, IL6, VEGF, and Arg1 in MDSC (194). Furthermore, TEX-associated Hsp72 triggers toll-like receptor (TLR)2/myeloid differentiation primary response gene 88 (MyD88)-dependent Stat3 activation in MDSC, which exert pronounced immunosuppressive activity (195). The finding was confirmed using MyD88-ko mice, which additionally revealed a reduction in CCL2 (196). TEX also promotes MDSC expansion in the BM through activation of STAT3, upregulated iNOS, which strengthens the immunosuppressive capacity of MDSC (197). Breast cancer-TEX distribute to the lung are taken up bone-marrow-derived cells and promote accumulation of MDSC in lung and liver. In addition, TEX inhibit through activation of M-MDSC T cell activation and TH1 cytokine production (198). BM stroma cell Exo, which are crucial in multiple myeloma development, are taken up by MDSC, induce their expansion, and survival through STAT3 and STAT1 pathway activation and induction of anti-apoptotic BclXl and Bcl2 family apoptosis regulator and promote NO release by MDSC increasing their suppressive activity on T cells (199).

Functional analysis of freshly *ex vivo* harvested MDSC-Exo was mostly restricted to the impact on myeloid cells. The authors report that the proinflammatory S100A8/9 heterodimer is chemotactic for MDSC (180). Furthermore, MDSC and, less prominently, MDSC-Exo convert tumoricidal M1–Mϕ to tumor growth-promoting M2-Mϕ by switching off IL12 production (180). Of special functional interest is the recovery of ubiquitinated histones and HMGB1 (182, 183), which exert proinflammatory activity, contribute to systemic inflammation and organ failure, and drive autoimmune diseases (200–202). HMBG1, a chaperone for many inflammatory molecules in MDSC, promotes the development of MDSC from BM progenitors, increases IL10 production by MDSC and contributes to downregulation of the T cell homing receptor CD62L (203, 204). The conversion of monocytes into MDSC-like cells and the differentiation of bone marrow cell into M-MDSC proceeds *via* the p38/NFκB/Erk1/2 pathway (205). In the context of chemoresistance, which in part relies on MDSC, MDSC-Exo miR-126a induces expansion of TH2, inhibits TH1 proliferation, and IFNγ secretion and supports angiogenesis. In a feedback loop, chemoresistance is transferred into the donor MDSC (193).

### Therapeutic Interference with MDSC-Exo in Cancer

There are excellent reviews on the therapeutic use of Exo (206, 207) as well as on attacking MDSC in cancer (208, 209) including approaches with a focus on improving the efficacy of immunotherapy (6, 210). So far, only a limited number of reports was concerned about directly attacking MDSC-Exo in cancer as a therapeutic option.

In brief, attacking tumor-infiltrating MDSC can be achieved by cytotoxic drugs, where ATRA blocks MDSC maturation (211, 212), which was explored in cancer immunotherapy (9, 20, 213) as well as in chronic infections (214). Gemcitabine particularly drives MDSC into apoptosis by a not yet fully explained mechanism (44), its efficacy in improving immunotherapy in cancer being repeatedly described (9, 47, 215). Sunitinib, a checkpoint signaling inhibitor, preferentially attacks MDSC and was reported in several tumor models to support immunotherapy (9, 216–218). Ongoing trials aim to increase vaccination efficacy by using combinations of drugs or radiotherapy (9, 219, 220).

Alternatively, an antibody blockade may be envisaged that prevents MDSC-Exo docking on target cells. According to the enrichment of tetraspanins, anti-CD9 was shown to prohibit breast cancer cell metastasis (221). We used anti-Tspan8 to block pancreatic cancer TEX, Tspan8 being abundantly expressed on pancreatic CSC-TEX (222). The antibody blockade sufficed to hamper angiogenesis and premetastatic niche establishment, but had a minor impact on MDSC (169). A MDSC-Exo selective antibody blockade remains to be explored.

An interesting approach is the use of proton pump inhibitors, toxic byproducts generated by the altered metabolism of cancer cells being expelled by proton transporters (223). Proton pump inhibitors concomitantly contributing to hamper the release of Exo by affecting the acid milieu in the tumor surrounding (224), the release of MDSC-Exo may be inhibited concomitantly, which could contribute to facilitate recruitment of effector immune cells. Similar considerations account for a blockade of the S100A8/9 marker on MDSC-Exo (180). Alternatively, a blockade of premetastatic niche formation was achieved by a blockade of CCL2 that prevented MDSC-Exo recruitment (225). A blockade of CD47 and its ligand Tsp and, less efficiently the signal regulator protein α, highly expressed on MDSC-Exo, also hampered MDSC-Exo chemotaxis and migration (185).

As an alternative approach, extracorporal hemofiltration is used for Exo elimination. Originally established as an affinity plasmapheresis for the elimination of TEX, it is being adapted to remove hepatitis C virions and is being explored to remove immunosuppressive Exo (226, 227). Progress in MDSC-Exo proteomics may provide means for a selective removal. There remains the problem of MDSC-Exo in cancer being mostly located within the tumor tissue or recruited to potential target, e.g., EC and premetastatic organ tissue rather than in the serum. Coping with Exo regeneration may also become demanding.

Last not least, Exo or Exo surrogates can be loaded with drugs, toxins, non-coding RNA to be delivered toward MDSC or MDSC-Exo to directly prohibit their immunosuppressive, angiogenesis and cancer-spread promoting activities (175, 178, 228, 229). The field is rapidly expanding, tailoring Exo, or surrogates also for repair, e.g., in artherosclerosis or thrombosis (230–232). There remains the demand for selective binding as, e.g., miRNA interfering with MDSC activities may promote tumor growth (189, 193, 233). Finally, great efforts are taken to replace Exo by nanoparticles that could allow for easier and homogeneous production (179). First trials attacking MDSC to improve cancer immunotherapy revealed encouraging results (234–237).

Thus, there are several promising options to interfere with the immunosuppressive and tumor growth-promoting activity of “inflammatory” MDSC and MDSC-Exo. There is need improving target selectivity. However, as the tumor milieu/TEX contribute to the recruitment and expansion of “inflammatory” MDSC/MDSC-Exo, targeting TEX may be considered under selected conditions as an alternative. Targeting TEX would be less demanding, as TEX mostly are equipped with oncogenes or CSC markers (222, 238, 239) that are not as widespread as inflammatory MDSC-Exo markers.

## Recruiting MDSC and MDSC-Exo in Autoimmune Disease

### MDSC and MDSC-Exo in Autoimmune Disease

While the abundance of MDCS/MDSC-Exo in cancer creates a milieu of therapy resistance, autoimmune disease progression is favored by the inefficacy of immune response regulation by immunosuppressive cells and factors (240). This accounts for the paucity of MDSC and Treg (240, 241), where the latter may be linked or be due to the former (242) and frequently is accompanied by an increase in TH17 (243). However, opposing findings were also reported.

Myeloid-derived suppressor cells only recently achieved attention in autoimmune diseases, initially in animal models such as experimental autoimmune encephalomyelitis (EAE), where a deficit in CCR2, which is required for MDSC recruitment, was accompanied by milder EAE (244). However, depending on the model and the readout system, opposing findings were also reported (245). This diversity of findings accounts for a wide range of studies on the recovery of MDSC and their suppressive activity in autoimmune diseases. There are, at least, two reasons for this confusion. First, the disease state is important. With progressive tissue destruction concomitantly to the dysregulated autoimmune effector cells, an inflammatory milieu is generated, which, in fact, supports MDSC activation. Second, a failure to detect a decrease in MDSC and/or Treg in the peripheral blood or peripheral lymphoid organs in autoimmune disease may be irrelevant (246), as the frequency in the autoimmune disease-affected organ can differ significantly. To give an example, while Treg are rare in the peripheral blood, in non-lymphoid tissues, the frequency of Treg ranges from 30–60% of the total CD4+ population (247).

Besides being concerned about the numeric MDCS/MDSC-Exo deficits, several studies elaborated a contribution of miRNA in their regulating. Thus, miR-181a is engaged in the maturation of myeloid progenitor cells, miR-17-5p, miR-20a, miR-106a, and miR-155 play a role in the differentiation of myeloid progenitor cells toward monocytes and miR-146a/b and miR-155 in the maturation toward Mϕ, where miR-155 and miR-181 additionally contribute to T cell differentiation (248). Monocytes in Sjoegren Syndrome show upregulated miR-34b-3p, -300, -609 expression; in psoriasis miR-223 was high and miR-193b was low in Th17 (249). In the antiphospholipid syndrome, which promotes trophoblast inflammation, changes in miR 146a-3p, -155, and -210 affected TLR signaling. These changes, also observed in MDSC-Exo, suggest a contribution to disease progression (250).

At the present state of knowledge, there is an urgent need for additional information on MDSC/MDSC-Exo presence and activity in autoimmune disease-affected organs. Instead, there is consent that chronic infections rely on an abundance of “inflammatory” MDSC/MDSC-Exo, which prevent appropriate activation of the adaptive and the innate immune system (251, 252). This knowledge, in fact, could provide a helpful guide toward MDSC/MDSC-Exo as a therapeutic option in autoimmune disease.

### MDSC and MDSC-Exo as a Therapeutic Option in Autoimmune Disease

There are excellent reviews on the link between chronic infections, immune regulation, and the associated hindrance of autoimmune disease development and progression (14, 39). MDSC/MDSC-Exo playing an important role, these inflammatory MDSC/MDSC-Exo may well provide a guide toward correcting overshooting reactions in autoimmune disease.

Thus, several reports demonstrating parasite infections being associated with a significant decrease in incidence or severity of immune diseases in animal models, the protective effect being due to Treg, alternatively activated Mϕ and changes in the cytokine profile (253–255). In chronic hepatitis C virus infection, a striking increase in M-MDSC was noted that expressed high level pSTAT3 and IL-10 and induced Treg expansion, where depletion of MDSC increased IFNγ production by CD4+ effector T cells (256). In human immunodeficiency virus-1 infections, too, MDSC-promoted Treg expansion and inhibited T cell function, a hallmark of chronic infections (257). In tuberculosis, the accumulation of MDSC prevented immune effector cell-mediated bacteria evasion (258). The interference of inflammatory MDSC/MDSC-Exo in cancer with immunotherapy was already outlined in detail.

As bacteria, parasites and viruses that cause chronic inflammation would rather provide a danger than a therapeutic option, chemical compounds that provoke delayed type hypersensitivity may be better suited to induce “inflammatory” MDSC. This option is well explored in alopecia areata (AA), most efficiently treated by the contact sensitizer squaric dibutylester (SADBE) (259, 260). SADBE treatment provokes a strong expansion of MDSC that inhibit autoreactive T cell activation and support Treg expansion. The effect is abolished by ATRA treatment (261). Notably, SADBE treatment can be replaced by the transfer of MDSC (262). In EAE, it was demonstrated that helminth products stimulate the production of TH2 cytokines and suppress TH1 and TH17 responses, the therapeutic efficacy exceeding that of corticosteroid treatment (263). Another option are statins, which are cholesterol lowering drugs, also described to induce immunosuppression. This was confirmed in acute and chronic dextran sodium sulfate (DSS)-induced colitis in mice, where statin-induced attenuation of colitis was due to expansion of MDSC (264).

Thus, the exploration of inflammatory MDSC has opened a path toward their therapeutic use in autoimmune disease. These studies clearly demonstrated therapeutic efficacy of MDSC in experimental autoimmune disease models (14, 265). In addition, good progress already was achieved replacing the infectious agents by synthetic compounds.

Autoimmune disease corrections by Exo, mostly by mesenchymal stem cell (MSC)–Exo, but also by DC-Exo were repeatedly described. To give a few examples, in diabetes susceptible mice, islet MSC release Exo that express endogenous retroviral antigens, which induce potent T and B cell responses (266). Application of MSC–Exo in experimental autoimmune uveitis exerted a therapeutic effect that was due to inhibiting the chemoattractive effects of CCL2 and CCL21 on inflammatory cells (267). Exo from miR-146a overexpressing DC suppress experimental myasthenia gravis by inducing an antigen-specific shift from TH1/TH17 to TH2/Treg (268). However, Exo from different donor cells or at different stages of disease may exert opposing activities. Thus, at early stages in chronic HBV infection, hepatic NK produce IFNγ in response to hepatic Mϕ. Hepatic Mϕ are stimulated by infected hepatocyte-Exo, which contain viral nucleic acids, *via* MyD88, toll-like receptor adaptor molecule (TICAM) and mitochondrial antiviral signaling protein to express NKG2D ligand. On the other hand, immunoregulatory miR-21 becomes upregulated in infected hepatocytes and is transferred *via* Exo in Mϕ suppressing IL12p35 expression, which counteracts the host innate immune response (269). For more comprehensive information, excellent reviews are recommended that outline the interplay between Exo from different donor cells and the activity of MDSC in autoimmune disease (270–272).

There is, to my knowledge, only one report explicitly describing the role of MDSC-Exo in autoimmune disease. Mice with DSS-induced colitis were treated with G-MDSC-Exo. G-MDSC-Exo sufficed for a significant decrease in disease severity and a reduction in the inflammatory cell infiltrate. TH1 cells were reduced and Tregs were augmented in the draining lymph node; in the serum IFNγ and TNFα were reduced. Inhibition studies pointed toward the impact of G-MDSC-Exo largely depending on Arg-1 (273).

Having described that the therapeutic efficacy of a chronic contact eczema in AA largely depends on the expansion of MDSC and that SADBE treatment can be replaced by MDSC application (261, 274), we proceeded controlling for the activity of MDSC-Exo in AA-affected mice. MDSC-Exo preferentially target *in vitro* and *in vivo* activated T cells, NK and most avidly Treg. Furthermore, an mRNA analysis of spleen cells of MDSC-Exo treated AA-affected mice showed a most striking increase in FoxP3 and Arg-1. These findings suggest MDSC-Exo strongly promoting Treg expansion and hampering innate immune reactions as well as T cell activation directly and *via* Treg.

Taking the knowledge collected in cancer and chronic infections on the power of inflammatory MDSC-Exo opened a path for a new wave of autoimmune disease treatment. Modalities to circumvent the potential danger of naturally arising inflammatory MDSC/MDSC-Exo have been suggested and are further explored in ongoing studies.

## Conclusion, Open Questions, and Outlook

**Figure 1:**
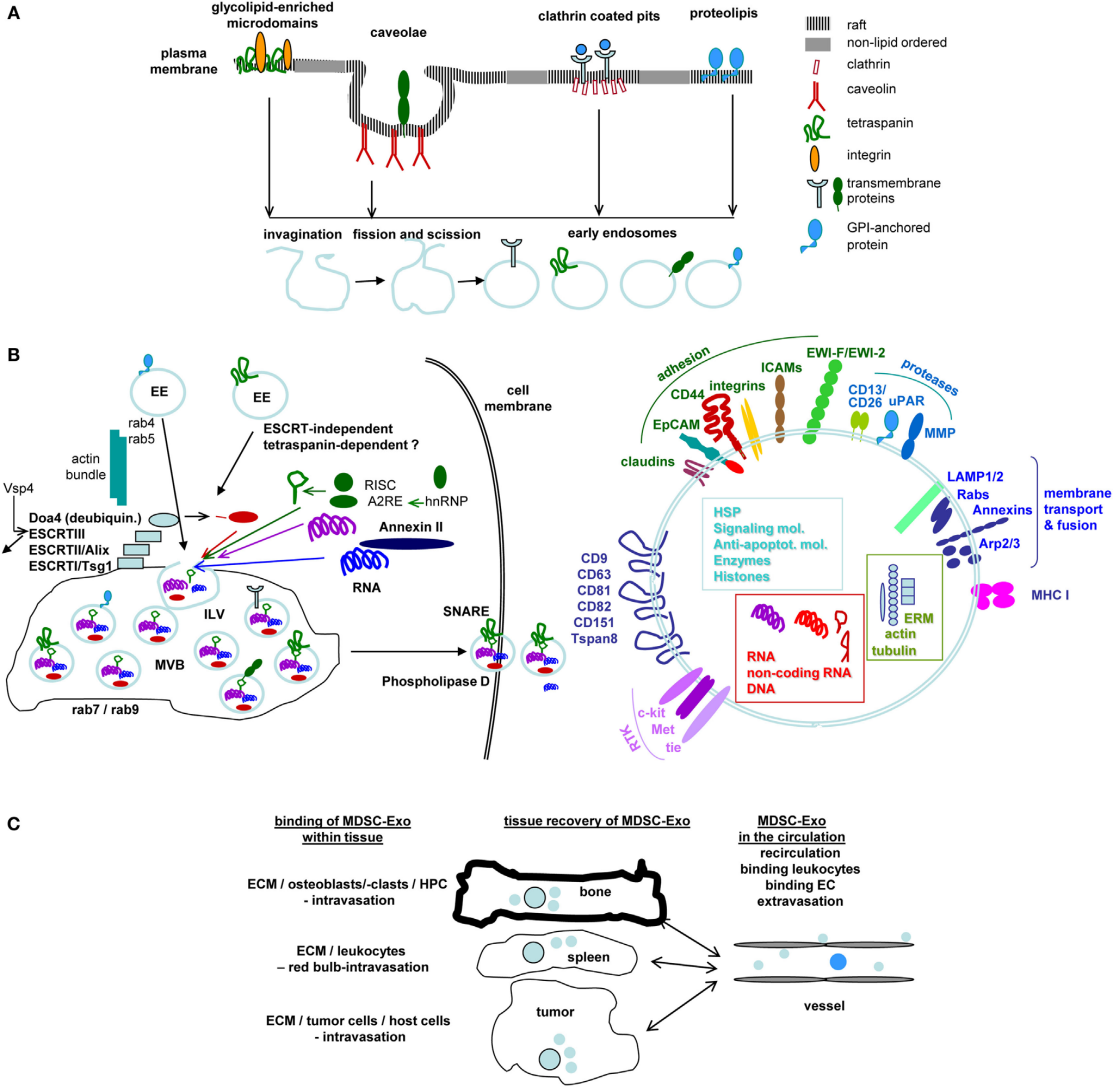

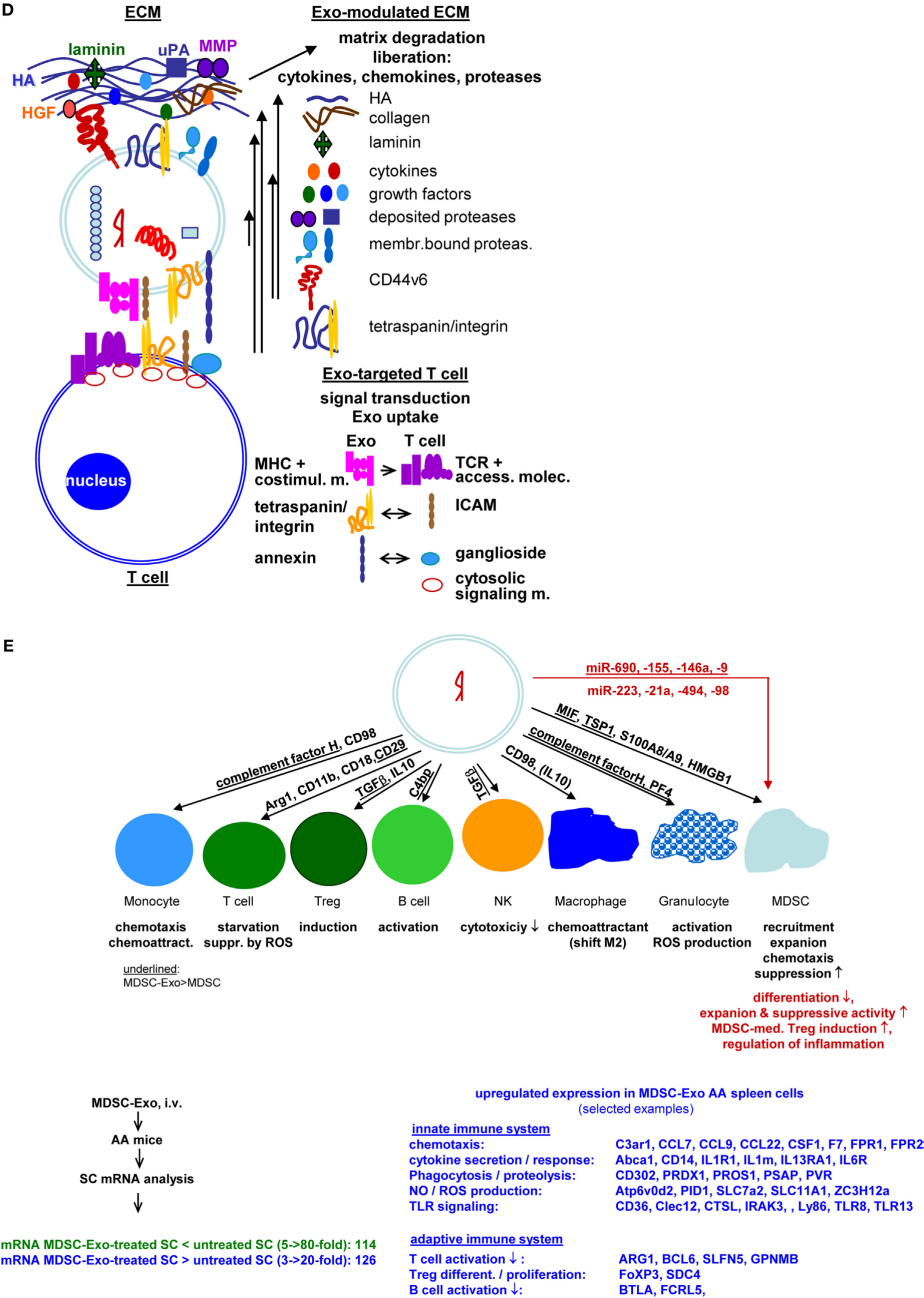
Exosomes (Exo) and therapy: open questions. **(A)** Exo derive from different membrane raft compartments, which are plane or invaginated, but are all prone for internalization due to enrichment for cholesterol and sphingolipids. Distinct lipid rafts harbor selective membrane-attached and transmembrane proteins, which are retained during invagination. This implies Exo derived from a single cell to be equipped by a different membrane coat. This poses the question on how to select for the appropriate myeloid-derived suppressor cells (MDSC)-Exo subpopulation. **(B)** After fission and scission, EE use different transporters toward MV, where ILV are loaded with proteins, coding and non-coding RNA and DNA during invagination into MVB. This is a selective process and includes components of different intracellular compartments. The abundance of selected molecules recruitment is only partly understood. Information is urgently required to judge on potential Exo activities. The released Exo are composed of the lipid membrane, the membrane-integrated and membrane-attached molecules, the components transferred into ILV and the majority of molecules engaged in vesicle transport, loading and Exo delivery. An arbitrarily selection is shown. (Alix: ALG-2 interacting protein X, Doad4: deubiquitinase, EE: early endosome, ESCRT: endosomal sortin complex, ILV, intraluminal vesicle; MVB, multivesicular body; SNARE, soluble-N-ethylmaleimide-sensitive fusion protein-attachment protein receptors; Vsp4, ATPase vacuolar protein sorting 4). **(C)** Exo distribute throughout the body and bind to matrix proteins and cells. Information on the availability of “free” Exo is limited. Yet, it is essential to judge on diagnostic and prognostic validity of Exo, including MSCD-Exo. **(D)** Exo binding to matrix proteins and cells are selective processes, where cells may use different ligands for binding and uptake. It is suggested that uptake depends on clustered ligands, possibly in invagination prone membrane domains. Binding, too, may be facilitated by clustered ligand. This is important for tailoring “therapeutic” Exo/Exo mimetics to facilitate binding/uptake or to prevent uptake. Selective examples are shown for ECM and T cell binding (ECM, extracellular matrix; HA, hyaluronic acid; HFG, hepatocyte growth factor; MHC, major histocompatibility complex; MMP, matrixmetallo proteinase; TCR, T cell receptor complex). **(E)** Exo binding and uptake modulates the target. Uptake initiated target cell modulation could proceed directly *via* incorporation of the Exo content, which recently was evaluated including miRNA (363) or by the target cell equipment after an initial hit by the Exo content. Both modalities were described. In view of the small Exo plasma and unpublished findings on changes in spleen cell mRNA after *in vivo* treatment of AA mice with MDSC-Exo, an initiating trigger may be more likely (AA, alopecia areata; Abca1, ATP-binding cassette sub-family A member 1; ARG1, Arginase-1; Atp6v0d2, V-type proton ATPase subunit d2; BCL, B cell leukemia; BTLA, B and T lymphocyte associated; CCL, chemokine ligand; C3ar1, C3a anaphylatoxin chemotactic receptor, Clec: C-type lectin domain family; MCSF1R, macrophage colony-stimulating factor 1 receptor; CTSL, Cathepsin L1; F7, coagulation factor VII; FCRL5, Fc receptor like 5; FOXP3, Forkhead box protein P3; FPR, fMet-Leu-Phe receptor; GPNMB, Transmembrane glycoprotein nMB; IL1R1, interleukin-1 receptor type 1; IL13RA1, IL13 receptor subunit alpha 1; IL6R, Interleukin6 receptor; IRAK, Interleukin-1 receptor-associated kinase, Ly, lymphocyte antigen; PID1, PTB-containing, cubilin and LRP1-interacting protein; SC, spleen cells; SDC, syndecan; SLC, Solute carrier family; SLFN5, Schlafen family member 5; TLR, toll-like receptor; ZC3H12a, zinc finger CCCH-type containing 12A). The nature of initiating triggers, target structures, and molecular pathways of progression remain to be defined. Clarification would greatly assist “therapeutic” Exo/Exo mimetic furnishing. Personal view: recovery of selected membrane markers of MDSC-Exo would be highly desirable. Should there be no selective markers, a binding unit for the target cell could be introduced. In concern about vesicle loading during biogenesis, the abundant recovery of proteasome subunits, histones, and splicing complex components requires special attention. It is conceivable that integration of these components rather than the small amount of transferred proteins, coding/non-coding RNA and DNA initiates target cell reprogramming by modulating transcription, translation, and metabolism. These activities will be well supported by MDSC-Exo binding to the T-cell and B-cell synapses, the receptor complexes and the adjacent accessory molecules being targeted by their counterparts on MDSC-Exo and being prone for internalization and initiation of signaling cascades. FcR and FcR-like molecules may cope with similar tasks in NK, granulocytes and Mϕ. Further progress in MDSC-Exo content elaboration and recovery in target cells will provide the answer, whether it appears more suitable loading MDSC-Exo with effector or initiator molecules.

- The discovery of Exo and other extracellular vesicles has revolutionized cell biology offering sessile cells to communicate over long distance (275). Though difficult to catch due to their heterogeneity (Figure 1A), where even a single cell delivers distinct Exo, great efforts are taken to answer open questions
  – We still poorly understand the process of loading the Exo plasma during biogenesis, including the enrichment of nuclear proteins, proteasome subunits, and components of the splicing machinery (Figure 1B).
  – The question of target molecules/complexes of target molecules is not comprehensively answered.
  – The availability of free versus bound/uptaken Exo requires further exploration (Figure 1C).
- MDSC, a heterogeneous population of immature myeloid cells, are important immune response regulators targeting T cells, B cells, Treg, NK, DC, and other cells of the innate immune system. This also accounts for MDSC-Exo, where target cells may become distinctly affected by MDSC binding versus MDSC-Exo uptake. MDSC/MDSC-Exo induce depletion of essential amino acids through ARG1, iNOS and IDO, NO, and ROS generation through iNOS and NOX2, anti-inflammatory cytokine production and Treg induction and activation (276). However, it is not well defined, whether
  – the equipment of MDSC/MDSC-Exo or of the target cell directs the mode of reprogramming.
  – MDSC and MDSC-Exo act *via* alike or different mechanisms.
- MDSC/MDSC-Exo are a major hindrance in tumor immunotherapy and chronic infections (39, 277, 278). Instead, autoimmune diseases may progress due to insufficient MDSC/MDSC-Exo (14, 19, 245). The inefficacy of MDSC/MDSC-Exo including the induction of Treg in autoimmune diseases is still debated. But, on site studies strongly support paucity of MDSC and Treg. Nonetheless,
  – the poor activation state of MDSC in the diseased tissue and
  – the mode whereby MDSC/MDSC-Exo provoke Treg expansion and activation requires further elaboration.
- The therapeutic efficacy of eliminating MDSC in cancer and of providing MDSC in autoimmune disease is well documented (44, 209, 245, 279). So far, only few reports were concerned about replacement by MDSC-Exo. However, MDSC-Exo are not only easier to handle, but also can be expected to act systemically and *via* their uptake to more severely affect their targets. The lack of information on selective markers of “inflammatory” MDSC-Exo provides a handicap. In cancer, MDSC recruitment and expansion are driven by TEX, which express cancer-related markers. Therefore, depletion of TEX instead of MDSC-Exo could provide an alternative. In concern of therapeutic MDSC-Exo substitution, “inflammatory” MDSC-Exo preferentially should be generated from synthetic compound stimulated MDSC, which avoids unwanted support of immunosuppression in response to naturally inflammatory stimuli. Irrespective of these alternatives,
  – the high prevalence of MDSC-Exo uptake by Treg and activated T cells suggests selective targets, which should be defined.
- This review focuses on MDSC-Exo and their activities in cancer and autoimmune disease. Nonetheless, the widespread activity particularly of SC-Exo (280) in physiology, including developmental patterning and the embryonic-maternal crosstalk (281, 282), in rejuvenation, regeneration, and repair (283) should, at least, be mentioned. SC-Exo act *via* signal transduction and the transfer of non-coding RNA (284, 285) and are suggested being a most potent therapeutics by maintaining stemness and inducing reparative programs (286, 287). There is justified hope on their therapeutic efficacy in SC transplantation, repair, and transplant acceptance (24, 288, 289).

Patrolling through the body to control for burglar and killers and to start the alarm clock was the privilege of cells of the innate immune system. For a long time, it was missed that they also control *via* Exo the response of the adaptive immune system, they had initiated. Taking into account that Exo are still newcomers in cell biology and all the excellent work collected during a short period, for which I apologize having cited only few, I am confident that open questions are quickly answered. This will provide a means to correct for overshooting and vanishing responses evolving in long-lasting diseases, such as cancer, chronic infections, and autoimmune diseases. The ease of tailoring Exo (290) will fortify therapeutic efficacy. Last, not least, provided open questions on Exo targeting and function-relevant components are answered (Figures 1D,E), Exo mimetics are expected to provide a homogeneous and reliably reproducible therapeutic agent (179, 291).

## Author Contributions

The author confirms being the sole contributor of this work and approved it for publication.

## Conflict of Interest Statement

The author declares that the research was conducted in the absence of any commercial or financial relationships that could be construed as a potential conflict of interest.

## Abbreviations

AA, alopecia areata; AGO, argonaute; Arg, arginase; ATRA, all-transretinoic acid; BclXl, Bcl2-like; BMC, bone marrow cells; BMSC, BM stroma cells; C, complement; CCL, C-C motif chemokine ligand; CEBP, CCAAT/enhancer binding protein; COX, cytochrome C oxidase; CSC, cancer stem cell; CXCL, C-X-C motif chemokine ligand; DC, dendritic cells; DSS, dextran sodium sulfate; EAE, experimental autoimmune encephalomyelitis; EC, endothelial cell; ECM, extracellular matrix; EMT, epithelial–mesenchymal transition; Dicer, ribonuclease; ESCRT, endosomal sorting complex required for transport; Exo, exosomes; Fbg, fibrinogen; FN, fibronectin; FOXO, forkhead box O; G-MDSC, granulocytic-MDSC; GM-CSF, granulocyte-macrophage-colony stimulating factor; GEM, glycolipid-enriched membrane domain; GPI, glycosylphosphatidylinositol; HCV, hepatitis C virus; HIF, hypoxia inducible factor; HIV, human immunodeficiency virus; HMBG, high motility group box; hnRNP, heterogeneous ribonucleoprotein; HO-1, heme oxygenase-1; Hp, haptoglobin; HSC, hematopoietic stem cells; HSP, heat shock protein; IDO, indoleamine 2,3-dioxygenase; ILV, intraluminal vesicles; iNOS, inducible nitric oxide synthase; IL, interleukin; IFN, interferon; JAK, Janus kinase; krt, keratin; LAMP, lysosomal-associated membrane protein; LETM1, leucine zipper and EF-hand containing transmembrane protein 1; LIPG, endothelial lipase; LN, laminin; lnc, long non-coding; LPS, lipopolysaccharide; MAVS, mitochondrial antiviral signaling protein; MCEMP1, mast cell expressed protein 1; Mcl1, Bcl2 family apoptosis regulator; M-CSF, macrophage colony-stimulating factor; Mϕ, macrophage; MDR, multidrug resistance gene; MDSC, myeloid-derived suppressor cells; M-MDSC, monocytic MDSC; MMP, metalloproteinase; MPO, myeloperoxidase; mTOR, mechanistic target of rapamycin; MSC, mesenchymal stem cells; MVB, multivesicular bodies; Myd88, myeloid differentiation primary response gene 88; NFκB, nuclear factor kappa B; NK, natural killer cell; NOXA, phorbol-12-myristate-13-acetate-induced protein 1; PBL, peripheral blood leukocytes; PG, prostaglandin; Pten, phosphatase and tensin homolog; Rab, ras-related proteins; RAGE, advanced glycosylation end-product specific receptor; RISC, RNA-induced silencing complex; ROS, reactive oxygen species; SADBE, squaric dibutylester; SC, stem cell; SCF, stem cell factor; Ship1, inositol phosphate-5-phosphatase D; SNARE, soluble-N-ethylmaleimide-sensitive fusion protein-attachment protein receptor; SOCS, suppressor of cytokine signaling; STAT, signal transducer and activator of transcription; TEX, tumor-derived Exo; TGF, transforming growth factor; TH, helper T cell; TLR, Toll-like receptor; TIM, T-cell immunoglobulin and mucin domain containing; TM9SF3, transmembrane 9 superfamily member 3; Treg, regulatory T cells; Tsp; thrombospondin; Tsg, tumor susceptibility gene; VEGF, vascular endothelial growth factor; VsP, ATPase vacuolar protein sorting.
